# Oral *Cannabis* consumption and intraperitoneal THC:CBD dosing results in changes in brain and plasma neurochemicals and endocannabinoids in mice

**DOI:** 10.1186/s42238-024-00219-x

**Published:** 2024-03-02

**Authors:** Nichole Reisdorph, Katrina Doenges, Cassandra Levens, Jon Manke, Michael Armstrong, Harry Smith, Kevin Quinn, Richard Radcliffe, Richard Reisdorph, Laura Saba, Kristine A. Kuhn

**Affiliations:** 1 Skaggs School of Pharmacy and Pharmaceutical Sciences, University of Colorado Anschutz Medical Campus, Aurora, CO 80045 USA; 2 School of Medicine, University of Colorado Anschutz Medical Campus, Aurora, CO 80045 USA

**Keywords:** Neurochemicals, Endocannabinoids, Edibles, Cannabis, THC, CBD, Cannabinoids, Mass spectrometry

## Abstract

**Background:**

While the use of orally consumed *Cannabis,* cannabidiol (CBD) and tetrahydrocannabinol (THC) containing products, i.e. “edibles”, has expanded, the health consequences are still largely unknown. This study examines the effects of oral consumption of whole *Cannabis* and a complex *Cannabis* extract on neurochemicals, endocannabinoids (eCB), and physiological parameters (body temperature, heart rate) in mice.

**Methods:**

In this pilot study, C57BL/6 J mice were treated with one of the following every other day for 2 weeks: a complex *Cannabis* extract by gavage, whole *Cannabis* mixed with nutritional gel through free feeding, or purified THC/CBD by intraperitoneal (i.p.) injection. Treatments were conducted at 4 doses ranging from 0–100 mg/kg/day of CBD with THC levels of ≤ 1.2 mg/kg/day for free feeding and gavage and 10 mg/kg/day for i.p. Body temperature and heart rate were monitored using surgically implanted telemetry devices. Levels of neurochemicals, eCB, THC, CBD, and 11-OH-THC were measured using mass spectrometry 48 h after the final treatment. Statistical comparisons were conducted using ANOVA and t-tests.

**Results:**

Differences were found between neurochemicals in the brains and plasma of mice treated by i.p. (e.g. dopamine, *p* < 0.01), gavage (e.g., phenylalanine, *p* < 0.05) and in mice receiving whole *Cannabis* (e.g., 3,4-dihydroxyphenylacetic DOPAC *p* < 0.05). Tryptophan trended downward or was significantly decreased in the brain and/or plasma of all mice receiving *Cannabis* or purified CBD/THC, regardless of dose, compared to controls. Levels of the eCB, arachidonoyl glycerol (2-AG) were decreased in mice receiving lowest doses of a complex *Cannabis* extract by gavage, but were higher in mice receiving highest doses compared to controls (*p* < 0.05). Plasma and brain levels of THC and 11-OH-THC were higher in mice receiving 1:1 THC:CBD by i.p. compared to those receiving 1:5 or 1:10 THC:CBD. Nominal changes in body temperature and heart rate following acute and repeated exposures were seen to some degree in all treatments.

**Conclusions:**

Changes to neurochemicals and eCBs were apparent at all doses regardless of treatment type. Levels of neurochemicals seemed to vary based on the presence of a complex *Cannabis* extract, suggesting a non-linear response between THC and neurochemicals following repeated oral dosing.

**Supplementary Information:**

The online version contains supplementary material available at 10.1186/s42238-024-00219-x.

## Introduction

The legal use of *Cannabis* products for medicinal and recreational use has expanded worldwide; however, critical research to understand the potential health consequences has not kept pace. The large number of *Cannabis* users, with a reported 22.2 million monthly U.S. users in 2015, includes those with various medical conditions, including mood disorders (Addiction CCoSU, n.d.; Aliev et al. 2020). This surge in *Cannabis* users has meant a concurrent expansion in the range of products that are available to consumers (Anderson et al. 2019). For example, a significant portion (~ 25%) of *Cannabis* consumers are utilizing “edibles” (Anderson et al. 2021), which can include gummies, chocolates, beverages, “full-spectrum” oils, and concentrated tinctures (Anderson et al. 2019; Barrus et al. 2016; Berg et al. 2023). These products contain various amounts of the psychoactive cannabinoid, delta-9-tetrahydrocannabinol (Δ9-THC, or generally referred to as THC), and the non-psychoactive cannabidiol (CBD). In a 2020 survey of 150 *Cannabis* retailers across 5 U.S. cities, edible potency ranged from 1 to 6% THC and 0.25 to 1 mg THC (Blum et al. 2021), with higher amounts of CBD providing a positive influence on purchasing decisions for both medicinal and recreational users (Blum et al. 2021; Borodovsky et al. 2016). The use of edibles compared to inhaled products is on the rise for several possible reasons: edibles do not contain combustible by-products, they are a more discreet form of THC and/or CBD consumption, and their use carries less stigma (Deiana et al. 2012).

All of the above-mentioned edible products may be prepared from whole *Cannabis*, complex *Cannabis* extracts, or with various concentrations of purified THC and CBD. Studies focusing on oral consumption of CBD- and THC-containing products is increasingly being conducted in both humans (Anderson et al. 2019; Deiana et al. 2012; Ewell et al. 2021) and animals (Friedman and Levin 2012; Galaj and Xi 2020). While more is becoming known about orally consumed CBD and THC, overall, several major gaps in knowledge exist and consumers currently are not informed about dosing, time to experience effects, and potential health consequences of oral *Cannabis* consumption (Aliev et al. 2020; Deiana et al. 2012; Gallily et al. 2015; Gallily and Yekhtin 2018). This includes understanding different effects due to product complexity and chemical composition (Geracioti et al. 2001).

Differences in both metabolism and psychotropic effects can be seen when orally dosing THC alone, with CBD, or as part of a complex *Cannabis* extract (Friedman and Levin 2012; Galaj and Xi 2020; Hermes et al. 2021). For example, when THC or CBD are given to rodents at a 1:1 ratio via intraperitoneal injection (i.p.), more THC is detectable in the brain compared to when THC is administered alone (Galaj and Xi 2020). Given that *Cannabis* contains well over 1,000 compounds including cannabinoids, terpenes, and flavonoids, it has been postulated that metabolism and effects of THC as part of orally administered complex plant extracts will depend at least in part on the chemical composition of the *Cannabis* preparation (Hlozek et al. 2017). There is also growing interest in understanding a possible "entourage effect" of complex formulations, whereby effects cannot be attributed to THC alone (Hermes et al. 2021; Koltai and Namdar 2020; Kosa et al. 2017). This is reflected in the marketplace in the number of edible products that contain THC in the form of complex *Cannabis* extracts, including products that have high CBD and low THC levels (Blum et al. 2021; Deiana et al. 2012). However, research on the metabolism and effects of orally consumed, complex *Cannabis* formulations is still lacking, in both humans and animal models. Therefore, one goal of this pilot study was to determine an appropriate dosing regimen of orally administered *Cannabis* and *Cannabis* extracts in mice.

While information on THC and CBD metabolism in both humans and animals is growing, the mechanisms behind the putative effects of THC, CBD and *Cannabis* on molecules related to mood disorders are still largely unknown. Previous research has demonstrated the effects of Δ9-THC on several neurochemicals, including dopamine, which has been previously implicated in mood disorders. In addition, evidence suggests that high doses of Δ9-THC increase dopamine release through Gamma-aminobutyric acid (GABA) activity and subsequent depressive qualities (Laprairie et al. 2015). In addition to altering levels of dopamine and GABA, Δ9-THC has been found to alter levels of other addiction-related neurochemicals, including glutamate and kynurenine (Leventhal et al. 2020). While effects of CBD on neurochemicals have not been as extensively studied as Δ9-THC, CBD is reported to affect the mesolimbic dopamine system and the serotonin 5-HT1A receptor system (McCarberg and Barkin 2007). Similarly, the amino acid tryptophan (Trp) is a critical component of numerous metabolic functions (McFall et al. 1990; Micale and Drago 2018; Newmeyer et al. 2016). Trp metabolism is most widely studied in relation to nervous system disorders and mental health (Obembe et al. 2015). Finally, phenylalanine (Phe) metabolism plays important roles in health and disease, predominantly through the generation of tyrosine and L-DOPA, epinephrine and norepinephrine, which have been linked to mood disorders (Okon et al. 2014a; Okon et al. 2014b; Panlilio and Justinova 2018). Therefore, a major goal of this research is to determine how specific neurochemicals are affected by oral consumption of *Cannabis*. This includes measuring both systemic (plasma) and local (brain) concentrations of over 20 neurochemicals including dopamine, norepinephrine, epinephrine, serotonin, glutamate, GABA, and kynurenine (Peng and Shahidi 2021).

THC and CBD have also been shown to affect the endocannabinoid system, which includes the endogenous cannabinoids (eCB), anandamide (ANA, also called arachidonoyl ethanolamide (AEA)) and arachidonoyl glycerol (2-AG), and cannabinoid-like lipid mediators such as palmitoyl ethanolamide (PEA) and oleoyl ethanolamide (OEA). These eCBs and lipid mediators can also act as endogenous ligands of CB1 receptors (Peters et al. 2021). To date, very few studies have examined changes in eCBs following THC exposure, either in adults or adolescents (Leventhal et al. 2020). Therefore, improving our understanding of the effects of *Cannabis* and THC on eCBs and neurochemicals related to mental health may facilitate identification of targets that can be used to treat these conditions, including mood disorders (Leventhal et al. 2020).

Overall, the complexity of *Cannabis* metabolism when orally consumed, and subsequent effects on neurochemicals and the eCB system, lends itself to initial studies in animals under highly controlled conditions to greatly inform our understanding of effects in humans. Therefore, the major goal of this pilot study was to determine if repeated oral administration of whole *Cannabis* and *Cannabis* extract resulted in changes to physiology, neurochemicals, and/or endocannabinoids in a mouse model. To evaluate changes in molecules related to mood disorders, both systemic (plasma) and local (brain) concentrations of over 20 neurochemicals (Peng and Shahidi 2021) and 14 eCBs were compared. Finally, changes in mouse physiology following initial and repeated exposure to *Cannabis* were evaluated using surgically implanted telemetry devices. In addition, to date, rodent studies have focused on acute dosing of THC and/or CBD. However, the current study design mimics real-world use of high CBD/low THC-containing edibles by humans, which are increasingly used by individuals to treat a variety of health issues (Addiction CCoSU, n.d.; Deiana et al. 2012; Reilly et al. 1997). Therefore, this pilot study aimed to examine sub-therapeutic to therapeutic doses of high CBD/low THC when fed to mice in the form of a complex *Cannabis* extract or whole *Cannabis*. We hypothesized that mice receiving *Cannabis* by any route would have increases in mood- and addiction-related neurochemicals such as dopamine and endorphins. Because the optimal dosing regimen required to elicit molecular and physiological changes following repeated oral dosing was not known at the time of study initiation, purified CBD/THC was also given via i.p. as a positive control. A key long term goal of this work is to provide insights on the effects of repeated oral dosing of *Cannabis* on dopamine, endocannabinoids, and related molecules.

## Methods

### Chemicals, standards, and reagents

Whole leaf *Cannabis sativa*, purified THC, and purified CBD were obtained from National Institutes of Drug Abuse (NIDA) under a DEA Schedule I license to Dr. Reisdorph. The Low THC (< 1%) / Very High CBD (> 10%) strain of *C. sativa* was utilized. Purified THC was provided as 10/mg/ml (-)-trans-Delta-9-THC in ethyl alcohol 95% and dried, synthetic CBD was reconstituted in 200 proof ethanol. Standards, chemicals and reagents for extraction and LC/MS analysis were of LC/MS or HPLC-grade. Ethanol (200 proof), Sodium Hydroxide (NaOH) and Sodium Chloride (NaCl) were purchased from Fisher Scientific (Fair Lawn, NJ). LC/MS water was purchased from Honeywell Burdick & Jackson (Muskegon, MI). Tween80 and sodium chloride was purchased from MilliporeSigma (St. Louis, MO). Cannabis plant material, THC and CBD standards were provided by NIDA (Bethesda, MD).

### Mice

**Fig. 1:**
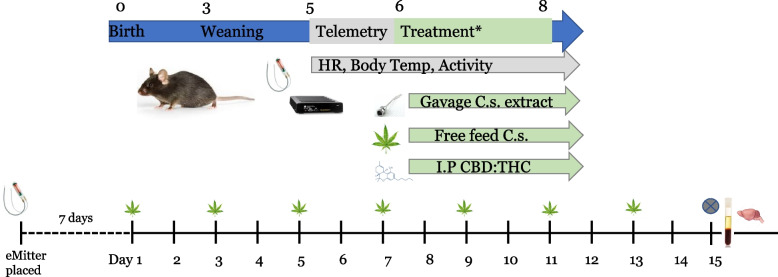
Schematic of experimental design. Following placement of telemetry devices in a subset of animals, mice were dosed every other day during the treatment phase (2 weeks). Dosing was conducted via gavage of a crude *Cannabis* extract, free feeding of whole *Cannabis*, or intraperitoneal injection (i.p.) of purified CBD/THC. Plasma and tissue were collected at study end

All mouse studies were approved by the University of Colorado Institutional Animal Care and Use Committee (IACUC). Male C57BL/6 J mice (Jackson Laboratories, Bar Harbor, ME) were maintained on a standard diet throughout the study. Mice were housed 1 per cage for mice receiving telemetry devices and no more than 2 per cage for mice not receiving telemetry devices. Mice were kept on regular 14 h/10 h light/dark cycle. Mice were weighed prior to and following placement of telemetry devices and on treatment days. Treatment began when mice were 8 weeks of age, with mice receiving treatments every other day for 2 weeks using dosing regimens described below and in Table 1. A minimum of 3 mice were included in each group with up to an additional 3 mice receiving telemetry devices. A full experimental design is shown in Fig. 1.

**Table 1:** Dosing regimen

Matrix	Delivery	Dosing Group	CBD mg/kg/day	Δ9-THC mg/kg/day
Purified THC:CBD	i.p	Control	0	0
Purified THC:CBD	i.p	Low	10	10
Purified THC:CBD	i.p	Medium	50	10
Purified THC:CBD	i.p	High	100	10
Crude Extract	Oral Gavage	Control	0	0
Crude Extract	Oral Gavage	Low	10	0.240
Crude Extract	Oral Gavage	Medium	20	0.480
Crude Extract	Oral Gavage	High	50	1.200
Whole *Cannabis*	Free feeding	Control	0	0
Whole *Cannabis*	Free feeding	Low	10	0.240
Whole *Cannabis*	Free feeding	Medium	20	0.480
Whole *Cannabis*	Free feeding	High	50	1.200

Mice received one of the following treatments (Matrix and Delivery): Purified CBD and THC via i.p., crude extract by oral gavage, or whole *Cannabis* mixed with nutritional gel by free feeding. The amount of CBD and THC in whole plant material was determined using mass spectrometry. Final dosing of CBD and THC at control, low, medium, and high concentrations are shown

### Selection of dosages

This pilot study was designed to determine if changes in neurochemicals, eCB, or physiology could be seen following repeated, oral dosing of high CBD/low THC in a whole *Cannabis* matrix in mice. Doses were based on the limited published reports that were available, to our knowledge, at study initiation, that utilized extracted *Cannabis (*Renard et al. 2017; Ressler and Nemeroff 2001; Richard et al. 2009) or purified THC/CBD (Roehler et al. 2022) specifically in mice. For example, Okon, et. al., administered 10 mg/kg-20 mg/kg per day of an organic extract of whole *Cannabis* buds for 28 days using oral gavage (Richard et al. 2009; Ruddick et al. 2006). Dosing in the study by Okon, et. al., was based on dry weight of *Cannabis* and phytocannabinoids concentrations were not measured. Dosing in a ratio of 10:1 CBD/THC was chosen based on the reported values of the NIDA whole *Cannabis* product. To our knowledge, at the time of study initiation, there had been no study that utilized whole, unextracted *Cannabis* for any type of intervention in mice; therefore, doses for whole *Cannabis* free feeding mimicked those used for gavage. Final concentrations of CBD/THC in the gavage and free fed mice were also limited by the volume or weight, respectively, of the final preparations that could be administered or consumed. Conversely, higher doses of THC and CBD administered in a single dose via i.p. had been shown to be safe in mice and hence higher doses were used for i.p. experiments (Roehler et al. 2022). The maximum dose of CBD was based on Deiana, et. al., who dosed animals with 120 mg/ml of purified CBD (Scherma et al. 2019).

### Mouse telemetry

Telemetry devices (eMitters, Starr Life Sciences) were surgically implanted in mice at age 6 weeks and mice were given 7–10 days to recover from surgery. Mice were anesthetized using inhaled continuous flow isofluorane (1–3% isoflurane) for the duration of the surgery (approximately 10 min). A small, 1 cm incision was made in the abdomen at the ventral midline and the transmitter placed under sterile conditions into the abdominal cavity of the mouse. The abdominal wall fascia was closed using simple interrupted 4–0 sterile absorbable suture. 100 µL of 0.025% bupivacaine was placed topically on the incision site and the skin layer was closed with sterile wound clips. Mice were removed from anesthesia and monitored for recovery for at least one hour. Post-operative pain control was provided SQ using Buprenorphine at a dose of 1 mg/kg one time. Mice were monitored twice daily over the following 72 h for mobility, wound closure, and general appearance. Staples/sutures were removed 7 days post-operatively. Transmitters remained in the mouse for the duration of the study. Following the 7 days recovery period, temperature and heart rate readings were recorded every minute.

### Preparation of C. sativa, C. sativa extracts, and CBD/THC

Approximately 250 mg of *Cannabis* was weighed into 16 × 125 mm borosilicate glass culture tubes (Fisher Scientific) fitted with PTFE/silicone-lined caps (VWR, Radnor, PA) and heat activated for 4 h at 120 °C on MULTIVAP Nitrogen Evaporator fitted with a heat block and culture tube adapters (Organomation, Berlin, MA). Aluminum foil was fitted over the heat block and tubes to retain heat. Activated *Cannabis* (50–150 mg) was weighed into TissueLyser tubes with a steel ball (Qiagen, Hilden, Germany). Tubes were centrifuged at room temperature and 18,000 × g for 2 min to prevent static movement of material. 200 proof ethanol was added to tubes at a ratio of 100 µL to 10 mg cannabis plant material. *Cannabis* was homogenized with a Qiagen TissueLyser LT bead mill for 5 min at 50 Hz and tubes were centrifuged at 0° C and 18,000 × g for 15 min. 20 µL of extract was reserved for targeted cannabinoid analysis in a 1.5 mL microcentrifuge tube at -20 °C. Remaining extract was used for mouse gavage.

### Mouse gavage

Gavage solutions were prepared under sterile conditions in a Nuair Biological Safety Cabinet (Plymouth, MN). Ethanol extracted *Cannabis* was sterile filtered in Pierce spin cups (Thermo Scientific, Waltham, MA) and centrifuged for 15 min at 4 °C and 18,000 × g. Flow through was pooled in a sterile 50 mL Falcon tube (Fisher Scientific), capped and vortexed well to mix. Twenty microliters of extract was reserved for targeted cannabinoid analysis in a 1.5 mL microcentrifuge tube and stored at -80° C until analysis.

Low, Medium and High doses (2.5, 5 and 12.5 mL, respectively) of pooled sterile-filtered extract were dried in 2 mL microcentrifuge tubes (Fisher Scientific) under nitrogen at 55 °C in 1–2 mL increments. The stock pooled extract was vortexed for 5 s before each addition to dose tubes. Dried extracts were reconstituted in 239.6 µL of ethanol and vortexed twice for 15 s to mix. A control dose was made from ethanol without *Cannabis* extract. Doses were sterile filtered in Pierce spin cups as above and stored at -80 °C. Dose solutions were made daily by combining the ethanol extracts with Tween80:3% saline solution (1:1:18 v/v/v) in 1.5 mL microcentrifuge tubes, stored at 4 °C and then transported on wet ice to the vivarium. Mice were gavaged at rates of 0, 10, 20 and 50 mg CBD/kg every other day for 2 weeks. Mice were weighed each morning prior to dosing. Dose solutions were vortexed well and approximately 100 µL was drawn up into 1 mL plastic syringe (Fisher Scientific) fitted with a 20 G × 38 mm plastic feeding tube (Instech Laboratories, Plymouth Meeting, PA). Feeding tubes were inserted through the mouth into the stomach of each mouse and the solution was administered.

### Mouse intraperitoneal injection

Intraperitoneal injection (i.p.) solutions were made under sterile conditions as above. A 200 mg powdered CBD standard was dissolved in 450 µL ethanol. Three 1,125 µL aliquots of the THC standard solution were combined with either 23.5, 117.2 or 234.4 µL of the CBD solution (Low, Medium and High dose, respectively) and evaporated to dryness in a Labconco CentriVap Concentrator (Kansas City, MO) at 45 °C. Dried residues were reconstituted in 225 µL ethanol and sterile filtered in Pierce spin cups (Thermo Scientific) as above. Mice were dosed at rates of 0, 10, 50 and 100 mg CBD/kg. THC concentrations were kept at 10 mg THC/kg, except in control group which was 0 mg/kg. Dose solutions were made fresh under sterile conditions by combining the ethanol dose solutions with the Tween80:3% saline mixture (1:1:18 v/v/v). Solutions were aliquoted daily, stored at 4 °C and then transported on wet ice to the vivarium. Dose solutions were vortexed well and approximately 100 µL was drawn up into 1 mL plastic syringe (Fisher Scientific) fitted with a 25 G needle and injected intraperitoneally.

### Free feeding

*Cannabis* plant material was heat activated and homogenized as described above, except without the addition of ethanol. One cube of bacon flavored Nutra-Gel (Bio-Serv, Flemington, NJ) was chilled at -20 °C for 15 min. Free feeding doses were prepared at 7.5, 10 and 30 mg/kg/day by combining appropriate amounts of *cannabis* (8, 10, 45 mg) and Nutra-gel (4.3, 4.3, 5 g, respectively) in a large weigh boat placed on ice. Doses were homogenized thoroughly by hand with two disposable 25 mm plastic cell scrapers (Fisher Scientific) and re-chilled at -20 °C for 15 min. Aliquots of 100 mg were weighed into 1.5 mL microcentrifuge tubes and stored frozen at -80 °C until day of dosing. Control doses were prepared in a similar manner, without the addition of *cannabis*. Daily dose aliquots were removed the morning of dosing and transported to the vivarium on wet ice. A 100 mm petri dish lid was placed into each mouse cage at the beginning of the study to administer the dose on. Mice were dosed every other day for two weeks. Mouse chow was removed from cages 2 h prior to free-feeding on dose days. Each dose aliquot was placed in the petri dish for easy access to the mouse. Mice were visually monitored to ensure complete ingestion and doses were typically consumed within 2 min.

### Dosing regimen

Two experiments were conducted to maximize the use of a limited number of telemetry devices (Table 1). Experiment 1 included a set of mice receiving crude *Cannabis* extract by gavage and a set of mice receiving purified CBD/THC by i.p. Experiment 2 included mice receiving whole *Cannabis* by free feeding and a second set of mice receiving purified CBD/THC by i.p. Dosing amounts of CBD and THC were based on published studies with the goal of administering non-toxic doses in the range of previously reported sub-therapeutic to therapeutic amounts of CBD (Ruddick et al. 2006; Schlienz et al. 2020). CBD and THC were measured in plant material and extracts using LC/MS/MS to confirm concentrations.

### Plasma and tissue collection

Mice were euthanized two days after the final treatment. Blood was collected in 1.3 mL K3EDTA micro sample tubes (Sarstedt Inc., Nuembrecht, Germany) via cheek bleeding, inverted 5 × to mix, and immediately placed on ice. Blood was centrifuged at 3,000 × g for 30 min at 4 °C, within 30 min of collection. Plasma was aliquoted into 1.5 mL microcentrifuge tubes and stored at -80 °C until analysis. Whole brains were collected in 15 mL Falcon tubes (Fisher Scientific), flash frozen in liquid nitrogen and stored at -80 °C until analysis.

### Phytocannabinoid, neurochemical and endocannabinoid analysis by liquid chromatography mass spectrometry

All standards and internal standards used for LC/MS/MS analysis of phytocannabinoids were purchased from Cerilliant (Round Rock, Texas, USA). Neurochemical standards and internal standards were acquired from various sources, including Cerilliant, Sigma-Aldrich (St. Louis, MO), Cayman Chemical (Ann Arbor, MI), and Cambridge Isotope Labs (Tewksbury, MA). All endocannabinoid standards were purchased from Cayman Chemical. All HPLC solvents and extraction solvents were HPLC grade or better. A list of all compounds included in the phytocannabinoid, neurochemical, and endocannabinoid assays is included in the Supplemental File 1. Only compounds found to be significant are included in the Results section; however, full results are available in the Supplemental Files 2–4.

#### Quantitation of cannabinoids in plant material and plant extracts

To assess extraction and filtration methods, a portion of the ethanol extract was analyzed prior to dosing mice. Portions of plant extractions were diluted by a factor of 10,000 in ethanol, transferred to a reduced surface activity/maximum recovery glass autosampler vial (Cornerstone Scientific, Wilmington, NC), and analyzed by LC/MS as described below.

#### Preparation of brain tissue homogenate

Brain samples were homogenized using a Qiagen TissueLyser LT (Qiagen, Hilden, Germany). Briefly, samples were placed into pre-chilled TissueLyser tubes containing a stainless-steel bead and a microliter volume of 1 × PBS solution equal to twice the tissue mass in milligrams was added to each sample. Samples were then homogenized at 50 Hz for 5 min followed by centrifugation for 10 min at 14,000 rpm and 4 °C. The supernatant was then collected and reserved for phytocannabinoid, neurochemical and endocannabinoid analysis.

#### Preparation of brain tissue and plasma for phytocannabinoid analysis

Proteins were precipitated from 25 µL of tissue supernatant or plasma by adding 25 µL of ice-cold methanol and 50 µL of the ice-cold internal standard solution (50 pg/µL each of Cannabidiol-D3, (-)-Δ9-THC-D3, ( ±)-11-Hydroxy-Δ9-THC-D3 in methanol) in a 1.5 ml microfuge tube, followed by vortexing and then incubating on ice for 15 min. The samples were then centrifuged for 10 min at 4 °C at 14,000 RPM and the supernatant was transferred to a reduced surface activity/maximum recovery glass autosampler vial for analysis.

#### Liquid chromatography mass spectrometry for phytocannabinoid quantitation

Quantitation of cannabinoids and neurochemicals was performed using reverse phase HPLC tandem mass spectrometry (LC/MS/MS). The HPLC system consisted of an Agilent 1290 autosampler (Agilent Technologies, Santa Clara, CA), an Agilent 1290 binary pump, and a 1200 series column compartment. Analysis buffers consisted of 0.1% formic acid in water (solvent A) and 1:1 acetonitrile: methanol (v:v, solvent B).

Two microliters of extracted plasma or 5 µL extracted brain was injected onto an Agilent Eclipse Plus C-18 2.1 × 50 mm 1.8 um analytical column with an Agilent SB C-18 2.1 × 5 mm, 1.8 um guard column using the following gradient at a flow rate of 0.4 mL/min: hold at 30% solvent A:70% solvent B from 0–4 min, then a linear gradient from 70–82% B over the next 3.5 min followed by an increase from 82–90% B from 7.5–8 min, then holding at 90% B for an additional 4 min. The analytical column was re-equilibrated at starting conditions for 3 min before the next injection.

Mass spectrometry analysis was performed on an Agilent 6490 triple quadrupole mass spectrometer in positive ionization mode. The drying gas was 120 °C at a flow rate of 12 mL/min. The sheath gas was 325 °C at 12 mL/min. The nebulizer pressure was 50 psi. The capillary voltage was 3500 V. Data for exogenous cannabinoids were acquired in dynamic MRM mode using experimentally optimized collision energies obtained by flow injection analysis of authentic standards (Supplemental File 1: Table 1). Calibration standards for each cannabinoid were analyzed over a range of concentrations from 0.5 – 10,000 pg on column. Calibration curves for each lipid mediator were constructed using Agilent MassHunter Quantitative Analysis software. Samples were quantitated using the calibration curves to obtain the on-column concentration, followed by multiplication of the results by the appropriate dilution factor to obtain the concentration in pg/ml. Levels of phytocannabinoids for several mice were below the method limit of quantitation (LOQ) and, therefore, results are expressed in peak areas.

#### Preparation of brain tissue and plasma for neurochemical analysis

Proteins were precipitated from 20 µL of brain tissue supernatant or plasma by adding 110 µL methanol and 10 µL of internal standard solution (Cambridge Isotope Laboratories, U13C metabolite yeast extract reconstituted in 3:1 ethanol:1 mM HEPEs pH 7.1 with adenosine-ribose-13C5 and creatinine-d3 added) in a 1.5 mL microfuge tube, followed by vortexing and then incubating at -20 °C for 10 min. The samples were then centrifuged for 10 min at 4 °C at 14,000 RPM and the supernatant was transferred to a reduced surface activity/maximum recovery glass autosampler vial for analysis.

#### Liquid chromatography mass spectrometry for neurochemical quantitation

Quantitation of neurochemicals was performed using Hydrophilic Interaction Liquid Chromatography (HILIC) HPLC tandem mass spectrometry (LC/MS/MS). The HPLC system consisted of an Agilent 1260 autosampler (Agilent Technologies, Santa Clara, CA), an Agilent 1260 binary pump, and a 1260 series column compartment. Buffer A consisted of 10 mM ammonium acetate adjusted to pH 9.0 with ammonium hydroxide, and buffer B consisted of 90:10 acetonitrile:water with 10 mM ammonium acetate adjusted to pH 9.0 with ammonium hydroxide. 5 µM of methylenediphosphonic acid was added to both buffers.

A total of 0.5 µL of extracted brain or plasma was injected onto an Agilent Poroshell 120 HILIC-Z 2.1 × 100 mm 2.7 um analytical column with the following gradient at a flow rate of 0.5 mL/min: hold at 100% solvent B for 2 min, 100%-80% solvent B from 2–12 min, 80%-60% solvent B from 12–13 min, hold at 60% B from 13–15 min, 60%-100%B from 15–16 min. The column was then re-equilibrated at 100%B for 4 min before the next injection.

Mass spectrometry analysis was performed on an Agilent 6490 triple quadrupole mass spectrometer in positive ionization mode. The drying gas was 130 °C at a flow rate of 15 mL/min. The sheath gas was 350 °C at 12 mL/min. The nebulizer pressure was 35 psi. The capillary voltage was 3000 V. Data for neurochemicals were acquired in dynamic MRM mode using experimentally optimized collision energies obtained by flow injection analysis of authentic standards (Supplemental File 1: Table 2). Calibration standards for each neurochemical was analyzed over a range of concentrations from 0.45– 235 pg on column for most of the target analytes, with epinephrine, metanephrine, normetanephrine, norepinephrine and 5-hydroxyindole-3-acetic acid from 4.5–2350 pg on column. Calibration curves for each neurochemical was constructed using Agilent MassHunter Quantitative Analysis software. Samples were quantitated using the calibration curves to obtain the on-column concentration, followed by multiplication of the results by the appropriate dilution factor to obtain the concentration in pg/mL or pg/mg.

#### Preparation of brain tissue and plasma for endocannabinoid analysis

Proteins were precipitated from 50 µL of brain tissue supernatant or plasma by adding 220 µL methanol and 30 µL of internal standard solution (Arachidonoyl ethanolamide-d4 and oleoyl ethanolamide at 200 pg/ µL, 2-arachidonoyl glycerol-d5 at 2000 pg/µL in methanol) in a 1.5 mL microfuge tube, followed by vortexing for ~ 10 s. The samples were then centrifuged for 10 min at 4 °C at 14,000 RPM and the supernatant was transferred to a reduced surface activity/maximum recovery glass autosampler vial for analysis.

#### Liquid chromatography mass spectrometry for endocannabinoid quantitation

Quantitation of endocannabinoids was performed using reverse phase HPLC tandem mass spectrometry (LC/MS/MS). The HPLC system consisted of an Agilent 1260 autosampler (Agilent Technologies, Santa Clara, CA), an Agilent 1260 binary pump, and a 1260 series column compartment. Buffer A consisted of 0.1% acetic acid in water and buffer B was 90:10 acetonitrile and isopropyl alcohol.

2 µL of extracted brain or plasma was injected onto an Agilent Eclipse Plus C18 2.1 × 150 mm 1.8um analytical column with the following gradient at a flow rate of 0.3 mL/min: 30%-45% solvent B from 0–2 min, 45%-79% solvent B from 2–2.5 min, hold at 79% solvent B from 2.5–11.5 min, 79% B-100% B from 11.5–12 min, hold at 100% B from 12–16 min, 100% B-30% B from 16–20 min. The column was then re-equilibrated at 30% B for 2 min before the next injection.

Mass spectrometry analysis was performed on an Agilent 6490 triple quadrupole mass spectrometer in positive ionization mode. The drying gas was 230 °C at a flow rate of 15 mL/min. The sheath gas was 400 °C at 11 mL/min. The nebulizer pressure was 35 psi. The capillary voltage was 4000 V. Data for endocannabinoids were acquired in dynamic MRM mode using experimentally optimized collision energies obtained by flow injection analysis of authentic standards (Supplemental File 1: Table 3). Calibration standards for each endocannabinoid were analyzed over a range of concentrations from 0.025– 50 pg on column for most compounds, with 2-linoleoyl glycerol at 0.25–500 pg on column and 2-arachidonoyl glycerol at 2.5–5000 pg on column. Calibration curves for each endocannabinoid were constructed using Agilent MassHunter Quantitative Analysis software. Samples were quantitated using the calibration curves to obtain the on-column concentration, followed by multiplication of the results by the appropriate dilution factor to obtain the concentration in pg/mL or pg/mg.

### Telemetry data processing

Processing of telemetry data was performed using Vital View software (Starr Life Sciences) according to the manufacturer’s suggestions. For temperature and heart rate data, missing values were first imputed by using the value just prior to the missing value. Following imputation, data were reduced by averaging values to obtain 6 data points per hour. Data were analyzed across time windows of 1 h increments, with windows designated as pre-dose, dose, post-dose 1, post-dose 2, post-dose 3, post-dose 4, post-dose 5, and post-dose 6. Therefore, a total of 8 h of data were considered.

### Statistics

For body weight analysis, a 2-way ANOVA was conducted (Prism GraphPad) for individual treatment groups. For phytocannabinoid analysis, a 1-way ANOVA followed by Tukey’s post-hoc analysis was used for pairwise comparisons between treatment groups (Prism GraphPad). For each neurochemical or endocannabinoid analyte, a 1-way ANOVA followed by Fischer’s LSD post-hoc analysis was used for pairwise comparisons between treatment groups (Prism GraphPad). For several of the neurochemical or endocannabinoid analytes, the overall p-value from the 1-way ANOVA was significant, but after a Tukey correction, none of the post hoc comparisons reached statistical significance. Because this is a pilot study, we chose to use a Fischer’s LSD post-hoc analysis instead of Tukey for the neurochemical or endocannabinoid analyte to better facilitate the identification of potential trends within the data. For heart rate and body temperature analyses, 2-way ANOVAs were conducted. A separate analysis of heart rate and body temperature of mice during the predose window confirmed no differences between groups prior to dosing ( *p* = 0.106).

## Results

### Brain and plasma levels of cbd, thc, and 11-oh-thc following treatment

Mice remained healthy throughout the study, with no noted changes in food or water intake, activity, or other health measures due to surgery or treatment. Mice receiving whole *Cannabis* or control via free feeding typically consumed the entire dose within 2 min. At study end, levels of CBD, THC, and 11-OH-THC in brain and plasma of mice were measured using LC/MS/MS. Samples were collected at 48 h following the final dose which resulted in some molecules being below the limit of quantitation of the mass spectrometer. For example, THC was only detected in one mouse receiving the high dose of *Cannabis* via free feeding; no other phytocannabinoids were detected in plasma or brain of mice treated by free feeding. Complete phytocannabinoid results can be found in Supplemental File 2.

**Fig. 2:**
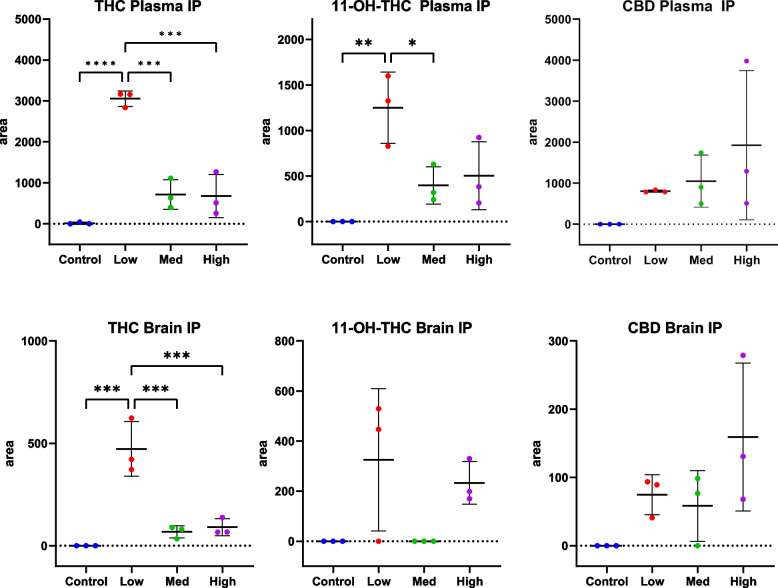
Significant changes (ANOVA *p* < 0.0001) in plasma levels of Δ9-THC and 11-OH-THC were determined following i.p. injection of THC/CBD (*n* = 3). Phytocannabinoids were measured in plasma using mass spectrometry at study end, approximately 48 h following the last dose. THC was dosed at 10 mg/kg/day for 14 days while CBD was given at 10 (1:1, Low), 50 (5:1, Medium), and 100 (10:1, High) mg/kg/day. As described in the methods, levels of phytocannabinoids for several mice were below the method limit of quantitation (LOQ) and, therefore, results are expressed in peak areas. Results for plasma phytocannabinoids are shown on the top graphs while results for brain phytocannabinoids are shown on the bottom graph. Significance thresholds are shown following Tukey post hoc analysis (*p < 0.05, **p < 0.01, *** *p* < 0.001)

For the i.p. treated animals from Experiment 1, ANOVA revealed significant differences in plasma levels (Fig. 2, top) of THC (ANOVA, *p* < 0.0001) and 11-OH-THC (ANOVA, *p* = 0.005) but not for CBD (ANOVA, *p* = 0.1878). As described in the methods, levels of phytocannabinoids for several mice were below the method limit of quantitation (LOQ) and, therefore, results are expressed in peak areas. For animals receiving treatment via i.p., CBD levels in plasma roughly demonstrated dose response, although plasma levels from animals receiving the high dose were variable. Results were more variable for CBD than for THC or 11-OH-THC. ANOVA showed differences for brain levels of THC (ANOVA, *p* < 0.001) but not for 11-OH-THC (ANOVA, *p* = 0.06) or CBD (ANOVA, *p* = 0.07) (Fig. 2, bottom) following i.p.. Both THC and CBD were detected in plasma from mice receiving the high dose by i.p. in Experiment 2; however, neither THC, CBD nor 11-OH-THC were measurable in mice receiving the low or medium doses or control animals.

For the gavage treated animals, ANOVA revealed differences in plasma levels of CBD (ANOVA, *p* = 0.0026), THC (ANOVA, *p* = 0.0023), and 11-OH-THC (ANOVA, *p* = 0.0382) (Supplemental Fig. 1). However, THC was only detected in 1 of the low dose mice and 11-OH-THC was only detected in 1 of the low dose and 1 of the medium dose mice. Therefore, no dose response was noted. In addition, only CBD was detected in the brains of gavage treated animals, and only in 2 of the 4 mice receiving the high dose. Neither THC nor 11-OH-THC were detected in the brains of gavage treated animals.

### Levels of brain and plasma neurochemicals following treatment

**Fig. 3:**
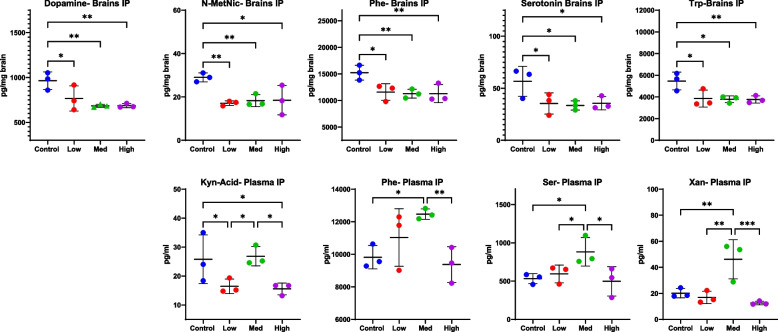
Significant changes in brain (top) and plasma (bottom) levels of neurochemicals were detected following i.p. injection of THC/CBD (*n* = 3–4). THC was dosed at 10 mg/kg/day for 14 days while CBD was given at 10 (1:1, Low), 50 (5:1, Medium), and 100 (10:1, High) mg/kg/day. Mass spectrometry was used to quantitate neurochemicals in brains and plasma of mice at study end. Trp = Tryptophan, Ser = Serotonin, Xan = Xanthurenic Acid, Ratios are of THC:CBD with THC at 10 mg/kg. Quinolinic acid was only detected in mice receiving i.p. THC/CBD (not shown). Significance thresholds are shown following ANOVA and uncorrected Fisher’s LSD post hoc analysis (*p < 0.05, **p < 0.01, ***p < 0.001)

Significant differences in brain and plasma levels of several neurochemicals were found between controls and all treatments of THC/CBD delivered via i.p. injection. For brain neurochemicals, ANOVA revealed significant differences in brain Trp (ANOVA, *p* = 0.0235), brain dopamine (ANOVA, *p* = 0.0125), brain N-MetNic (ANOVA, *p* = 0.0172), and brain Phe (ANOVA, *p* = 0.0235) (Fig. 3). For these neurochemicals, higher levels were seen in controls compared to all doses (Fig. 3), with no dose response noted. Nominal differences were seen for brain Ser (ANOVA, *p* = 0.0554). While no significant differences were found when ANOVA was used to compare levels of L-Kyn, post-hoc analysis determined differences between control and high dose for L-Kyn (*p* = 0.0303, Fisher’s LSD post-hoc analysis) (Supplemental File 3). However, for plasma Xan (ANOVA *p* = 0.0038), ANOVA revealed differences between CBD:THC i.p. doses, with highest levels in mice receiving the medium dose (Fig. 3). Similarly, significant differences in plasma Ser (ANOVA, *p* = 0.0486), plasma Phe (ANOVA, *p* = 0.0359) and plasma Kyn (ANOVA, *p* = 0.0372)(Fig. 3) were disclosed using ANOVA, with higher levels in mice receiving medium doses of cannabinoids. Quinolinic acid was only detected in mice receiving the high dose of THC/CBD via i.p. (Supplemental File 3).

**Fig. 4:**
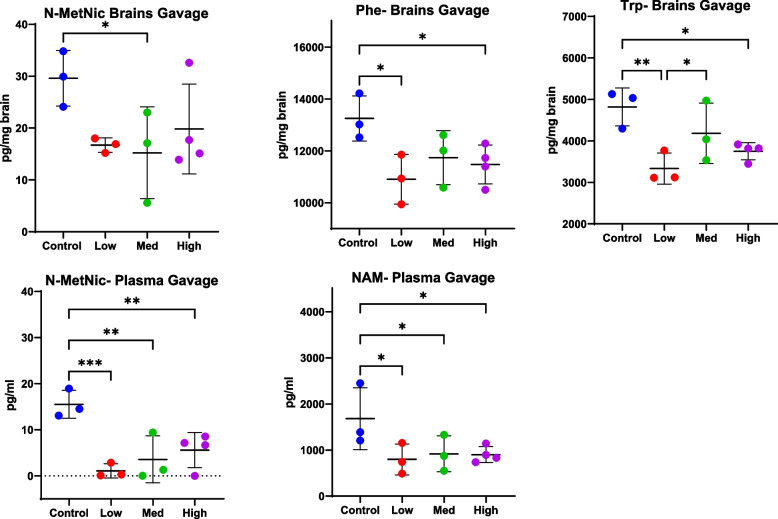
Significant changes in brain (top) and plasma (bottom) levels of neurochemicals were detected following gavage of a complex *Cannabis* extract (*n* = 3,4). Mass spectrometry was used to quantitate neurochemicals in brains and plasma of mice at study end. Mice received either vehicle control or crude *Cannabis* extract by gavage at the following doses of CBD and THC, respectively: 10 and 0.240 mg/kg/day (low), 20 and 0.480 mg/kg/day (medium), or 50 and 1.2 mg/kg/day (high). See also Table 1. Trp = Tryptophan, Phe = Phenylalanine, NAM = Niacinamide, N-MetNic = N-methylnicotinamide. Significance thresholds are shown following ANOVA and uncorrected Fisher’s LSD post hoc analysis (*p < 0.05, **p < 0.01, ***p < 0.001)

ANOVA also revealed differences between some neurochemicals in the brains of animals treated by gavage (ANOVA, Phe *p* = 0.0515, Trp *p* = 0.0166) and in the plasma (ANOVA, N-MetNic *p* = 0.0043) (Fig. 4). In these gavage-treated animals, some changes trended in a dose dependent manner; for example, N-MetNic appeared somewhat higher in animals receiving higher doses by gavage although only nominal or no significance was reached following post hoc analysis (Control vs. Low *p*-value = 0.05; Control vs Med *p*-value = 0.03; Control vs High p-value 0.10, Fisher’s LSD post-hoc analysis). No dose-dependent effects were seen for either brain Phe or Trp, with only differences between control and mice receiving the low dose being noted following post-hoc analysis (Phe Cont vs Low *p*-value = 0.01; Phe Control vs High *p*-value 0.03; Trp Control vs Low *p*-value = 0.003; Trp Control vs High *p*-value = 0.01; Trp Low vs Medium p-value 0.05, Fisher’s LSD post-hoc analysis). No differences in plasma NAM were noted when ANOVA was used for analysis (ANOVA, *p*-value = 0.09) but significance was reached between control and all 3 doses following post-hoc analysis (*p* < 0.05, Fisher’s LSD post-hoc analysis). In addition, differences were seen in brain kynurenic acid (Kyn) when medium and high doses were compared following post-hoc analysis (*p* = 0.0357, Fisher’s LSD post-hoc analysis) and in N-MetNic when control and medium doses were compared (*p* = 0.0334, Fisher’s LSD post-hoc analysis) (Supplemental File 3).

**Fig. 5:**
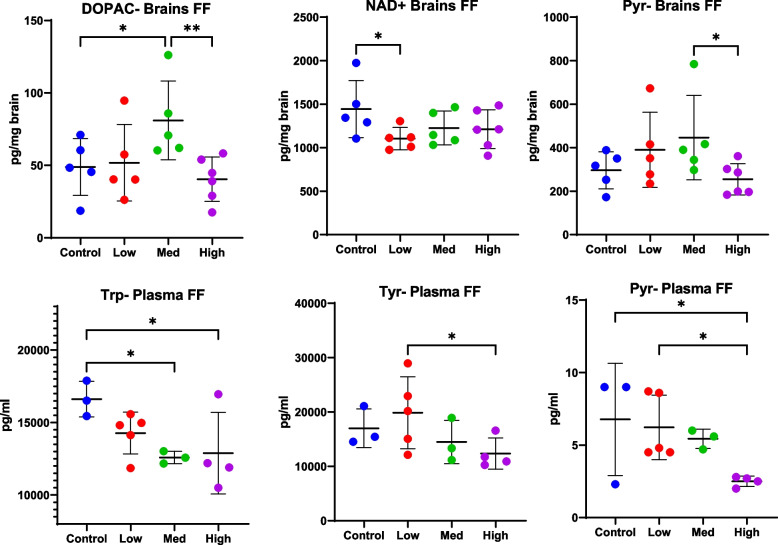
Changes in brain (top) and plasma (bottom) levels of neurochemicals were detected following free feeding of whole *Cannabis* (*n* = 5, 6). Mass spectrometry was used to quantitate neurochemicals in brains and plasma of mice at study end. Mice were presented either Nutragel control or whole *Cannabis* in Nutragel at the following doses of CBD and THC, respectively: 10 and 0.240 mg/kg/day (low), 20 and 0.480 mg/kg/day (medium), or 50 and 1.2 mg/kg/day (high). See also Table 1. Mice consumed the food in under 10 min. DOPAC = 3,4-Dihydroxyphenylacetic acid, NAD + = Nicotinamide adenine dinucleotide, Pyr = Pyridoxyal, Trp = Tryptophan, Tyr = Tyrosine. See Table 1 for dosing. Significance thresholds are shown following ANOVA and uncorrected Fisher’s LSD post hoc analysis (*p < 0.05, **p < 0.01)

Finally, nominal changes in neurochemicals were detected with free feeding mice whole *Cannabis* in a NutraGel food matrix where the doses were identical to those treated by gavage (Fig. 5). However, levels for only one neurochemical, brain DOPAC, passed significance following ANOVA (ANOVA, *p* = 0.0467). Rather, in the free fed animals, changes were noted between specific doses following post hoc analysis. For example, levels of plasma Tyr were higher in mice receiving the low dose compared to those receiving high dose (*p* = 0.0414, Fisher’s LSD post-hoc analysis). Levels of brain NAD + were lower in control compared to low dose (*p* = 0.0321, Fisher’s LSD post-hoc analysis). Similar to the other treatment regimens, Trp trended down in the plasma of all animals receiving *Cannabis* by free feeding (ANOVA, *p* = 0.0615) although significance was found only when low and medium doses were compared to controls (*p*-values < 0.05, Fisher’s LSD post-hoc analysis). Differences in the vitamin B co-factor, pyridoxal, were found for brain, when medium and high doses were compared (*p* = 0.351, Fisher’s LSD post-hoc analysis) and for plasma, when control vs high (*p* = 0.249, Fisher’s LSD post-hoc analysis) and medium vs high (*p* = 0.0257, Fisher’s LSD post-hoc analysis) were considered. However, while plasma levels trended towards a decrease in mice consuming *Cannabis*, brain levels were only higher in mice receiving low and medium doses compared to control (Fig. 5- right most panels).

### Changes in endocannabinoids and ethanolamides (eCB system) following treatment

We also determined if changes in endocannabinoids and related ethanolamides occurred in plasma and/or brains of study mice. For these experiments, the eCBs 2-AG and AEA, and 11 related molecules, including DHEa, DEA, LEA, OEA, and SEA were measured using a validated LC/MS/MS assay (Sharma et al. 2012). Complete results are found in Supplemental File 4.

**Fig. 6:**
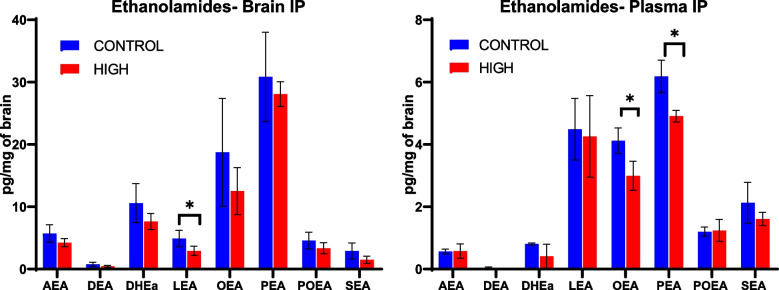
Significant changes (*p* < 0.05) in several eCB-like lipid molecules were detected in brains (left) and plasma (right) of mice following i.p. injection of THC/CBD (*n* = 5). 2-AG levels trended downward in mice receiving THC/CBD (not shown). THC was dosed at 10 mg/kg/day for 14 days while CBD was given at 10 (1:1, Low), 50 (5:1, Medium), and 100 (10:1, High) mg/kg/day. Mass spectrometry was used to quantitate ethanolamides in brains and plasma of mice at study end. Ethanolamide abbreviations: AEA-Arachidonoyl, DEA = Docosatetraenoyl DHEa = Docosahexaenoyl, LEA = Linoleoyl, OEA = Oleoyl, PEA = Palmitoyl, POEA = Palmitoleoyl, SEA = Stearoyl

Due to sample volume limitations, eCBs were only measured in mice receiving i.p. in Experiment 2, where only control and high doses were used. Levels of LEA were found to be significantly lower (t-test, *p* < 0.05) in brains of mice receiving THC/CBD via i.p. compared to controls (Fig. 6). While several other molecules trended downward in treated versus control mice, no other significant differences were found. Levels of PEA and OEA were significantly lower (t-test, *p* < 0.05) in plasma of these same mice.

**Fig. 7:**
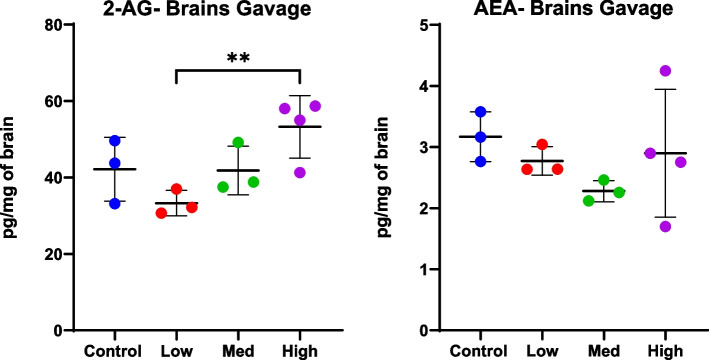
Levels of the eCBs 2-AG and AEA in brains of animals orally dosed with *Cannabis* extract by gavage. Mass spectrometry was used to quantitate eCBs in brains and plasma of mice at study end. Mice received either vehicle control or crude *Cannabis* extract by gavage at the following doses of CBD and THC, respectively: 10 and 0.240 mg/kg/day (low), 20 and 0.480 mg/kg/day (medium), or 50 and 1.2 mg/kg/day (high). See also Table 1. High doses resulted in lower AEA but were variable and did not reach significance

Levels of 2-AG showed an interesting pattern in brains of mice receiving *Cannabis* extract by gavage, with a decrease in mice receiving lowest doses (0.24 mg Δ9-THC) compared to controls, but higher levels in mice receiving highest doses (1.2 mg Δ9-THC) compared to controls (ANOVA *p* < 0.05, Fig. 7). Levels of AEA also trended lower in the brains of mice receiving low and medium doses of gavage compared to control, although neither ANOVA nor post-hoc analyses revealed differences when all treatment groups were used for analysis (Fig. 7).

**Fig. 8:**
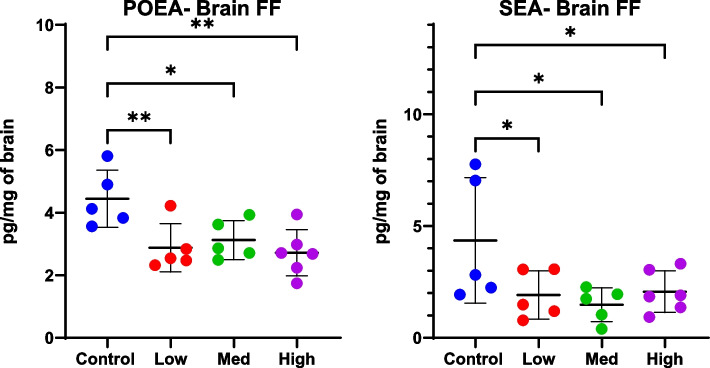
Brain levels of the eCB-related molecules, POEA and SEA, were significantly different between controls and mice free fed whole *Cannabis* at almost any dose (ANOVA *p*-value = 0.04). Concentration of CBD and THC given per kg/day were, respectively as follows: Low = 10 and 0.24, Medium = 20 and 0.48, and High = 50 and 1.2 mg. Significance thresholds are shown following ANOVA and uncorrected Fisher’s LSD post hoc analysis (*p < 0.05, **p < 0.01)

ANOVA revealed differences in levels of POEA and SEA in the brains of mice free fed *Cannabis* (ANOVA, POEA *p* = 0.008, SEA *p* = 0.044) with significantly lower levels in control mice compared to all treatment doses following post hoc analysis (Fig. 8; *p*-values < 0.05, Fisher’s LSD post hoc analysis). Post-hoc analysis also revealed differences in other eCB-like molecules; for example, DEA was lower in the mice fed low and medium doses compared to control (*p* < 0.05, Fisher’s LSD post hoc analysis) (Supplemental File 4).

### Changes in mouse physiology following treatment

Temperature and heart rate data obtained from surgically implanted telemetry devices were analyzed on the first and last treatment days to evaluate acute effects following initial and repeated exposure to *Cannabis*. As described in the methods, telemetry devices recorded body temperature and heart rate each minute and data were processed to generate an average of 6 readings per hour. Data were analyzed across time windows of 1 h increments, with windows designated as pre-dose, dose, post-dose 1, post-dose 2, post-dose 3, post-dose 4, post-dose 5, and post-dose 6. Therefore, a total of 8 h of data were considered. As expected, no significant differences in temperature or heart rate of mice were seen in any treatment groups when the pre-dose windows were compared prior to treatment (i.e. mice were the same on day 1, prior to treatment).

#### Body temperature

**Fig. 9:**
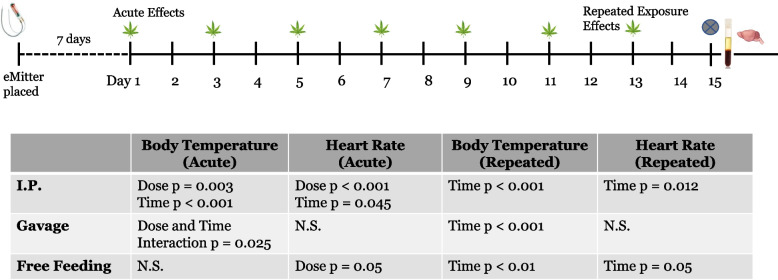
Changes in body temperature and heart rate following acute and repeated exposure. Temperature and heart rate data obtained from surgically implanted telemetry devices were analyzed on the first (Acute) and last (Repeated) treatment days to evaluate acute effects following initial and repeated exposure to *Cannabis*. Telemetry devices recorded body temperature and heart rate each minute and data were processed to generate an average of 6 readings per hour. Data were analyzed across time windows of 1 h increments, with windows designated as pre-dose, dose, post-dose 1, post-dose 2, post-dose 3, post-dose 4, post-dose 5, and post-dose 6. Therefore, a total of 8 h of data were considered. Two-way ANOVA was conducted

Comparison of 1-h time windows on the first day of treatment was used to examine acute effects on body temperature during initial exposure to *Cannabis*. Acute effects were seen with i.p. (dose *p* = 0.03 and time *p* < 0.001) but the effect of time on body temperature did not differ between doses (Fig. 9). Time and Dose-dependent, acute effects were seen with gavage (interaction effect *p* = 0.025, Fig. 9). No significant effects on body temperature during initial exposures were seen when mice were free-fed *Cannabis*. Overall, changes in body temperature appeared to occur at the later time points and at similar levels in all groups (Fig. 9 and Supplemental File 1: Fig. 2).

Similarly, the acute effects on body temperature following repeated exposure to *Cannabis* was evaluated by comparing 1 h time windows on the final day of dosing. Effects were seen for all doses of i.p (*p* < 0.001) and gavage (*p* < 0.001) when time windows were compared (Fig. 9). However, no dose effects were noted. Significant differences in body temperatures of mice free-fed *Cannabis* were found via two-way ANOVA when time windows were compared (*p* = 0.01), but this was not dose dependent.

#### Heart rate

The same strategy of comparing 1 h time windows on the first day of treatment to evaluate effects of initial exposure to *Cannabis* used to analyze the heart rate data. There were significant main effects on heart rate of both dose (*p* < 0.001) and time window (*p* = 0.045) when mice received THC/CBD by i.p., but the interaction effect was not significant (*p* = 0.15) (Fig. 9). No significant effects on heart rate during initial exposures were seen when mice received *Cannabis* extract via gavage. Conversely, nominally dose-dependent effects on heart rate were noted for mice free-fed *Cannabis* (*p* = 0.05).

Similarly, the acute effects on heart rate following repeated exposure to *Cannabis* was evaluated by comparing 1 h time windows on the final day of dosing. When mice received THC/CBD via i.p., a difference across time windows is suggested (*p* = 0.012) although that difference is consistent across dosage group (i.e. not significant, *p* = 0.112) (Fig. 9) when two-way ANOVA is used. No significant effects on heart rate following repeated exposures were seen when mice received *Cannabis* extract via gavage. A nominal time window effect of heart rate (*p* = 0.05) is seen in the free-fed mice; however, that effect does not differ between dosage groups (i.e., the interaction effect is not significant). Therefore, the time window estimates were calculated both within each dosage group and between dosage groups. The only time window contrast between Pre Dose and each of the other time windows that was significant (unadjusted *p*-value < 0.05) was Post dose 4.

## Discussion

This pilot study aimed to examine sub-therapeutic to therapeutic doses of high CBD/low THC when orally administered to mice in the form of a complex *Cannabis* extract or whole *Cannabis*. In addition, one group of mice received purified THC:CBD via i.p. to mimic higher doses CBD/THC and hence serve as a positive control. To our knowledge, this is one of the first animal studies that aimed to determine how specific neurochemicals in the tryptophan and dopamine pathways are affected by oral consumption of *Cannabis*. We hypothesized that oral administration of *Cannabis* would result in increases in mood-related neurochemicals such as dopamine. We also expected to detect phytocannabinoids in plasma and brains of mice who had received THC and CBD by any form.

Both Δ9-THC and its first pass metabolite, 11-OH-THC, were quantified in the plasma and brain of all animals treated via i.p. at 48 h following the final treatment. Interestingly, plasma and brain levels of Δ9-THC and 11-OH-THC were higher in animals receiving 1:1 THC:CBD by i.p. compared to those receiving 1:5 or 1:10 THC:CBD (Fig. 2, top showing results in plasma). These data are consistent with others who have shown differences in the metabolism of Δ9-THC when administered alone compared to with CBD via i.p. (Galaj and Xi 2020).

For animals receiving treatment via i.p., CBD levels in plasma roughly demonstrated dose response, although plasma levels from animals receiving the high dose were variable. THC and 11-OH-THC levels were higher in mice receiving the low dose of CBD compared to those receiving medium and high doses of CBD. In this study, THC was dosed at 10 mg/kg/day, while CBD were 0, 10, 50, and 100 mg/kg/day; therefore, it appears that mice receiving lower doses of CBD had the highest levels of THC. Others have postulated this could be due to competition between THC and CBD (Galaj and Xi 2020).

For mice receiving a complex *Cannabis* extract by oral gavage, Δ9-THC and 11-OH-THC were present at variable levels in both plasma and brain (Supplemental File 1: Fig. 1 and Supplemental File 2). Levels of CBD in gavage animals was roughly dose responsive, with much higher levels in animals receiving higher dose. Overall, the few studies that have measured plasma Δ9-THC concentration following oral administration of *Cannabis* to mice have demonstrated variable results, likely due to differences in the composition of the food matrix, which influences rate of Δ9-THC absorption from the gut (Anderson et al. 2019; Deiana et al. 2012; Spindle et al. 2020; Taylor et al. 2019). Because plasma and brain were collected 48 h following the final dose, some molecules were not detected in all samples; this is not surprising since it is now known that Δ9-THC has largely been metabolized by this time (Galaj and Xi 2020). For example, Δ9-THC was only detected in one mouse receiving the high dose of *Cannabis* via free feeding; no other cannabinoids were detected in plasma or brain of mice treated by free feeding.

Previous research has demonstrated the effects of Δ9-THC on several neurochemicals, including dopamine, which has been previously implicated in mood disorders. In the current study, mice receiving CBD/THC via i.p. had decreased dopamine compared to control animals, although this response was not dose dependent (Fig. 3). Levels of dopamine in free fed or gavaged mice did not change significantly although dopamine was detected in the vast majority of samples from these mice (Supplemental File 3). However, changes were noted in the dopamine metabolite, 3,4-dihydroxyphenylacetic acid (DOPAC) in mice orally consuming the medium versus high dose (Fig. 5). In addition, the precursor to dopamine, phenylalanine, was significantly reduced in the brains of mice receiving i.p. and gavage treatment (Figs. 3 and 4); however, levels were variable in plasma of mice receiving i.p. treatment. Δ9-THC exerts its effects as a partial agonist of Cannabinoid 1 (CB1) and, to a lesser effect, CB2 receptors which are part of our endogenous cannabinoid system (Todd et al. 2017; Uziel et al. 2020; Vandrey et al. 2017). The binding of Δ9-THC to CB1 leads to increased dopamine release with subsequent, powerful reward and reinforcement messages, that may alleviate pain and discomfort but may also lay a foundation for addiction (Volkow et al. 2014). However, regular *Cannabis* use, which is more similar to the current study, results in reduced dopamine response in the brain (Volkow et al. 2014). Overall, the results from the current study support previous work showing that repeated *Cannabis* use may result in overall dampened dopamine metabolism (Weiss 2015).

In addition to altering levels of dopamine, Δ9-THC has been found to alter levels of other mood disorder and addiction-related neurochemicals, including glutamate and kynurenine (Kyn) (Leventhal et al. 2020). In the current study, some neurochemicals in the Trp/Kyn pathway, including brain Trp, brain Ser, and brain n-Met-NAC, were decreased in i.p. treated mice; however, no dose response was noted. Since levels of CBD increased while levels of Δ9-THC remained at 10 mg, this suggests that Δ9-THC is driving changes in these molecules rather than CBD. However, for plasma molecules in this pathway, such as plasma Xan, plasma Ser and plasma Phe (Fig. 3), differences between CBD:Δ9-THC doses were noted. A dose difference was also seen in plasma N-MetNic in gavaged mice. This suggests a non-linear effect of Δ9-THC when Δ9-THC is given in combination with another cannabinoid or in a complex extract. Similar to the effects noted for the Phe/Dopamine pathway, overall there appears to be a down-regulation of the Trp/Kyn pathway.

Similar to the other treatment regimens, Trp trended downward in all animals receiving whole *Cannabis* by free feeding; i.e., no significant dose response was seen with Trp. This could be a result of flux to 1 of 4 Trp metabolic pathways in response to stress or stimulus, namely to serotonin, kynurenine, proteins, or indoles. The relationship between *Cannabis*, the eCB system, and stress is beginning to be explored (Zhu et al. 2021). Conversely, Tyr was highest in the plasma of control free fed animals while brain pyridoxal was highest in mice fed the medium dose. DOPAC was also highest in the brains of animals free-fed the medium dose while NAD + was down in the brains of all animals free-fed *Cannabis*. This further supports the idea that levels of neurochemicals can vary based on the presence of a complex *Cannabis* extract and suggests a non-linear response between Δ9-THC and neurochemicals.

In addition to neurochemicals, Δ9-THC has also been shown to affect both endogenous cannabinoids, AEA and 2-AG, and cannabinoid-like lipid mediators such as PEA and OEA. In the current study, treatment with THC/CBD via i.p. significantly reduced eCBs in both brain and plasma (Fig. 6). Similarly, levels of POEA and SEA in the brains of free fed mice decreased with treatment. Conversely, levels of 2-AG in the brains of gavage mice appeared to decrease with the low dose but increase with the high dose. In rats, repeated exposure to Δ9-THC alone resulted in lower levels of AEA and 2-AG in the striatum (Leventhal et al. 2020); these 2-AG results differ from the current study, where a complex *Cannabis* extract was used. Another study demonstrated that Δ9-THC-induced increases in dopamine release could be interrupted by disrupting eCB signaling (Laprairie et al. 2015). Therefore, increasing eCBs could be a therapeutic alternative for patients suffering from CUD by alleviating withdrawal symptoms or could act as a replacement therapy prior to attaining CUD. Finally, drugs blocking 2-AG or AEA transport or metabolism are currently being investigated as possible treatments for addiction and neuropathic pain (see (Leventhal et al. 2020) for review).

Overall, these initial experiments demonstrate changes to eCBs following treatment with Δ9-THC:CBD by i.p. or oral administration of Δ9-THC via a complex *Cannabis* extract and whole *Cannabis*. Because eCBs also bind the CB1 and CB2 receptors, our findings suggest a mechanism whereby Δ9-THC may result in decreased levels of eCBs. The endocannabinoids 2-AG and AEA are produced by 2 different metabolic pathways, and there appears to be an inverse relationship between the two molecules in this study. To our knowledge, the mechanism(s) by which Δ9-THC can upregulate enzymes in the 2AG pathways (PLC and DAG lipase) and downregulate the AEA pathways (NAT and NAPE-PLD) is not currently known.

While administration of purified THC:CBD by i.p.resulted in changes in neurochemicals and eCB, only subtle changes in body temperature and heart rate were found in mice acutely and repeatedly dosed with THC:CBD via i.p. Acute changes were significant for both dose and time separately while only a time effect was seen for body temperature and heart rate following repeated dosing. Significant changes in body temperature, but not heart rate, were seen after acute and repeated dosing of a crude *Cannabis* extract by gavage. Conversely, there were no changes to body temperature in mice free fed *Cannabis* on day 1 (acute). However, significant differences in heart rate were seen after acute and repeated free feeding. Together, these data suggest effects on physiology following acute treatments are dose- and time-dependent whereas dose has less of an effect when mice are repeatedly exposed to *Cannabis*.

This pilot study had several limitations that raise caution in interpreting results. The overall sample sizes per treatment group (*n* = 3–6) and sample volumes were sufficient for the measurement of neurochemicals and eCB molecules; however, high variability in some treatment groups suggest even larger sample sizes are necessary. In addition, larger sample sizes are required for fully evaluating telemetry results. Pharmacokinetic studies had not been conducted in mice at the time of study initiation, to our knowledge, that focused on repeated oral administration of *Cannabis*. However, a study conducted in fasted rats showed small levels of cannabinoids remained in blood and brain 24 h following subcutaneous administration of purified CBD + THC (Galaj and Xi 2020). Since no studies focused on repeated oral administration of *Cannabis* or a complex *Cannabis* extract to mice in an unfasted state, the PK profiles were unknown. While cannabinoids were detectable in some samples, they were not measured consistently in samples from mice that were free fed *Cannabis*. For this pilot study, initial doses were based on achieving non-toxic, sub-therapeutic to therapeutic CBD and THC doses in a whole *Cannabis* matrix. While some reports have shown lower bioavailability of phytocannabinoids when orally consumed, a study by Hlozek, et. al. showed that orally consumed THC/CBD had better absorption compared to subcutaneous and pulmonary administration. While orally dosed mice in the current study had some changes in neurochemicals, endocannabinoids, and physiology, it is likely that higher doses are needed to obtain more significant results. Finally, the doses of up to 100 mg/kg CBD and 10 mg/kg CBD used in this study are much higher than typical human consumption of a THC-containing edible. For example, a typical recommendation for edibles is 10 mg THC, which for a human weighing 150 pounds (68 kg) is 0.15 mg/kg. Therefore, while the lower gavage and free fed doses are within this range, the i.p. doses are pharmacological dosages that exceed what would be expected in human consumption.

## Conclusions

A generalizable protocol for the dosing of mice using whole *Cannabis* plant and a complex *Cannabis* extract is demonstrated. Results suggests a non-linear effect of Δ9-THC on neurochemicals when Δ9-THC is given in combination with another phytocannabinoid or in a complex extract. Overall, this pilot study provides a foundation for future studies that can further evaluate oral consumption of *Cannabis*, CBD, and/or Δ9-THC in the context of specific disease and/or behavioral models.

### Supplementary Information

**Supplementary Material 1.**

**Supplementary Material 2.**

**Supplementary Material 3.**

**Supplementary Material 4.**

## Data Availability

The datasets used and/or analyzed during the current study are either included in this published article and/or supplementary files or are available from the corresponding author on reasonable request.

## Abbreviations

DOPAC: 3,4-Dihydroxyphenylacetic acid
ANA: Anandamide
AEA: Arachidonoyl ethanolamide
2-AG: Arachidonoyl glycerol
Δ9-THC or THC: Delta-9-tetrahydrocannabinol
CBD: Cannabidiol
DEA: Docosatetraenoyl
DHEa: Docosahexaenoyl
Kyn or L-Kyn: Kynurenine
LEA: Linoleoyl
Phe: Phenylalanine
L-DOPA: 3,4-Dihydroxyphenylalanine
OEA: Oleoyl ethanolamide
PEA: Palmitoyl ethanolamide
Ser: Serotonin
SEA: Stearoyl
Trp: Tryptophan
Tyr: Tyrosine
Xan: Xanthurenic Acid

## References

[CR1] Addiction CCoSUa. 7 things you need to know about edible Cannabis [Available from: https://www.ccsa.ca/sites/default/files/2019-06/CCSA-7-Things-About-Edible-Cannabis-2019-en.pdf.

[CR2] Aliev G, Beeraka NM, Nikolenko VN, Svistunov AA, Rozhnova T, Kostyuk S (2020). Neurophysiology and Psychopathology Underlying PTSD and Recent Insights into the PTSD Therapies-A Comprehensive Review. J Clin Med..

[CR3] Anderson LL, Low IK, Banister SD, McGregor IS, Arnold JC (2019). Pharmacokinetics of Phytocannabinoid Acids and Anticonvulsant Effect of Cannabidiolic Acid in a Mouse Model of Dravet Syndrome. J Nat Prod.

[CR4] Anderson LL, Etchart MG, Bahceci D, Golembiewski TA, Arnold JC (2021). Cannabis constituents interact at the drug efflux pump BCRP to markedly increase plasma cannabidiolic acid concentrations. Sci Rep.

[CR5] Barrus DG, Capogrossi KL, Cates SC, Gourdet CK, Peiper NC, Novak SP, et al. Tasty THC: Promises and Challenges of Cannabis Edibles. Methods Rep RTI Press. 2016;2016. 10.3768/rtipress.2016.op.0035.1611 PMC5260817 28127591

[CR6] Berg CJ, Romm KF, Pannell A, Sridharan P, Sapra T, Rajamahanty A (2023). Cannabis retailer marketing strategies and regulatory compliance: A surveillance study of retailers in 5 US cities. Addict Behav.

[CR7] Blum K, Khalsa J, Cadet JL, Baron D, Bowirrat A, Boyett B (2021). Cannabis-Induced Hypodopaminergic Anhedonia and Cognitive Decline in Humans: Embracing Putative Induction of Dopamine Homeostasis. Front Psychiatry.

[CR8] Borodovsky JT, Crosier BS, Lee DC, Sargent JD, Budney AJ (2016). Smoking, vaping, eating: Is legalization impacting the way people use cannabis?. Int J Drug Policy.

[CR9] Deiana S, Watanabe A, Yamasaki Y, Amada N, Arthur M, Fleming S (2012). Plasma and brain pharmacokinetic profile of cannabidiol (CBD), cannabidivarine (CBDV), Delta(9)-tetrahydrocannabivarin (THCV) and cannabigerol (CBG) in rats and mice following oral and intraperitoneal administration and CBD action on obsessive-compulsive behaviour. Psychopharmacol.

[CR10] Ewell TR, Abbotts KSS, Williams NNB, Butterklee HM, Bomar MC, Harms KJ (2021). Pharmacokinetic Investigation of Commercially Available Edible Marijuana Products in Humans: Potential Influence of Body Composition and Influence on Glucose Control. Pharmaceuticals (Basel)..

[CR11] Friedman M, Levin CE (2012). Nutritional and medicinal aspects of D-amino acids. Amino Acids.

[CR12] Galaj E, Xi ZX (2020). Possible Receptor Mechanisms Underlying Cannabidiol Effects on Addictive-like Behaviors in Experimental Animals. Int J Mol Sci..

[CR13] Gallily R, Yekhtin Z, Hanuš LO (2015). Overcoming the Bell-Shaped Dose-Response of Cannabidiol by Using Cannabis Extract Enriched in Cannabidiol. Pharmacol & Pharma.

[CR14] Gallily R, Yekhtin Z (2018). Avidekel Cannabis extracts and cannabidiol are as efficient as Copaxone in suppressing EAE in SJL/J mice. Inflammopharmacol..

[CR15] Geracioti TD, Baker DG, Ekhator NN, West SA, Hill KK, Bruce AB (2001). CSF norepinephrine concentrations in posttraumatic stress disorder. Am J Psychiatry.

[CR16] Hermes DJ, Yadav-Samudrala BJ, Xu C, Paniccia JE, Meeker RB, Armstrong ML (2021). GPR18 drives FAAH inhibition-induced neuroprotection against HIV-1 Tat-induced neurodegeneration. Exp Neurol.

[CR17] Hlozek T, Uttl L, Kaderabek L, Balikova M, Lhotkova E, Horsley RR (2017). Pharmacokinetic and behavioural profile of THC, CBD, and THC+CBD combination after pulmonary, oral, and subcutaneous administration in rats and confirmation of conversion in vivo of CBD to THC. Eur Neuropsychopharmacol.

[CR18] Koltai H, Namdar D (2020). Cannabis Phytomolecule 'Entourage': From Domestication to Medical Use. Trends Plant Sci.

[CR19] Kosa KM, Giombi KC, Rains CB, Cates SC (2017). Consumer use and understanding of labelling information on edible marijuana products sold for recreational use in the states of Colorado and Washington. Int J Drug Policy.

[CR20] Laprairie RB, Bagher AM, Kelly ME, Denovan-Wright EM (2015). Cannabidiol is a negative allosteric modulator of the cannabinoid CB1 receptor. Br J Pharmacol.

[CR21] Leventhal AM, Bae D, Kechter A, Barrington-Trimis JL (2020). Psychiatric comorbidity in adolescent use and poly-use of combustible, vaporized, and edible cannabis products. J Psychiatr Res.

[CR22] McCarberg BH, Barkin RL (2007). The future of cannabinoids as analgesic agents: a pharmacologic, pharmacokinetic, and pharmacodynamic overview. Am J Ther.

[CR23] McFall ME, Murburg MM, Ko GN, Veith RC (1990). Autonomic responses to stress in Vietnam combat veterans with posttraumatic stress disorder. Biol Psychiatry.

[CR24] Micale V, Drago F (2018). Endocannabinoid system, stress and HPA axis. Eur J Pharmacol.

[CR25] Newmeyer MN, Swortwood MJ, Barnes AJ, Abulseoud OA, Scheidweiler KB, Huestis MA (2016). Free and Glucuronide Whole Blood Cannabinoids' Pharmacokinetics after Controlled Smoked, Vaporized, and Oral Cannabis Administration in Frequent and Occasional Cannabis Users: Identification of Recent Cannabis Intake. Clin Chem.

[CR26] Obembe AO, Okon VE, Ofutet EO, Okpo-ene AI (2015). Effect Of Chronic Consumption of Cannabis Sativa on Bleeding Time, Prothrombin Time and Platelet Count In Albino Rats. Int J Sci Res.

[CR27] Okon VE, Obembe AO, Nna VU, Osim EE (2014). Long-Term Administration of Cannabis sativa on Locomotor and Exploratory Behavior in Mice. Res Neurosci.

[CR28] Okon VE, Obembe AO, Nna VU, Osim EE (2014). Long Term Administration of Cannabis sativa Reduces Food, Water Intake and Body Weight in Mice. Int J Sci Res.

[CR29] Panlilio LV, Justinova Z (2018). Preclinical Studies of Cannabinoid Reward, Treatments for Cannabis Use Disorder, and Addiction-Related Effects of Cannabinoid Exposure. Neuropsychopharmacol.

[CR30] Peng H, Shahidi F (2021). Cannabis and Cannabis Edibles: A Review. J Agric Food Chem.

[CR31] Peters KZ, Oleson EB, Cheer JF (2021). A Brain on Cannabinoids: The Role of Dopamine Release in Reward Seeking and Addiction. Cold Spring Harb Perspect Med..

[CR32] Reilly JG, McTavish SF, Young AH (1997). Rapid depletion of plasma tryptophan: a review of studies and experimental methodology. J Psychopharmacol.

[CR33] Renard J, Norris C, Rushlow W, Laviolette SR (2017). Neuronal and molecular effects of cannabidiol on the mesolimbic dopamine system: Implications for novel schizophrenia treatments. Neurosci Biobehav Rev.

[CR34] Ressler KJ, Nemeroff CB (2001). Role of norepinephrine in the pathophysiology of neuropsychiatric disorders. CNS Spectr..

[CR35] Richard DM, Dawes MA, Mathias CW, Acheson A, Hill-Kapturczak N, Dougherty DM (2009). L-Tryptophan: Basic Metabolic Functions, Behavioral Research and Therapeutic Indications. Int J Tryptophan Res.

[CR36] Roehler DR, Hoots BE, Holland KM, Baldwin GT, Vivolo-Kantor AM (2022). Trends and characteristics of cannabis-associated emergency department visits in the United States, 2006–2018. Drug Alcohol Depend.

[CR37] Ruddick JP, Evans AK, Nutt DJ, Lightman SL, Rook GA, Lowry CA (2006). Tryptophan metabolism in the central nervous system: medical implications. Expert Rev Mol Med.

[CR38] Scherma M, Masia P, Satta V, Fratta W, Fadda P, Tanda G (2019). Brain activity of anandamide: a rewarding bliss?. Acta Pharmacol Sin.

[CR39] Schlienz NJ, Spindle TR, Cone EJ, Herrmann ES, Bigelow GE, Mitchell JM (2020). Pharmacodynamic dose effects of oral cannabis ingestion in healthy adults who infrequently use cannabis. Drug Alcohol Depend.

[CR40] Sharma P, Murthy P, Bharath MM (2012). Chemistry, metabolism, and toxicology of cannabis: clinical implications. Iran J Psychiatry.

[CR41] Spindle TR, Cone EJ, Herrmann ES, Mitchell JM, Flegel R, LoDico C (2020). Pharmacokinetics of Cannabis Brownies: A Controlled Examination of Delta9-Tetrahydrocannabinol and Metabolites in Blood and Oral Fluid of Healthy Adult Males and Females. J Anal Toxicol.

[CR42] Taylor M, Cousijn J, Filbey F (2019). Determining Risks for Cannabis Use Disorder in the Face of Changing Legal Policies. Curr Addict Rep.

[CR43] Todd SM, Zhou C, Clarke DJ, Chohan TW, Bahceci D, Arnold JC (2017). Interactions between cannabidiol and Delta(9)-THC following acute and repeated dosing: Rebound hyperactivity, sensorimotor gating and epigenetic and neuroadaptive changes in the mesolimbic pathway. Eur Neuropsychopharmacol.

[CR44] Uziel A, Gelfand A, Amsalem K, Berman P, Lewitus GM, Meiri D (2020). Full-Spectrum Cannabis Extract Microdepots Support Controlled Release of Multiple Phytocannabinoids for Extended Therapeutic Effect. ACS Appl Mater Interfaces.

[CR45] Vandrey R, Herrmann ES, Mitchell JM, Bigelow GE, Flegel R, LoDico C (2017). Pharmacokinetic Profile of Oral Cannabis in Humans: Blood and Oral Fluid Disposition and Relation to Pharmacodynamic Outcomes. J Anal Toxicol.

[CR46] Volkow ND, Wang GJ, Telang F, Fowler JS, Alexoff D, Logan J (2014). Decreased dopamine brain reactivity in marijuana abusers is associated with negative emotionality and addiction severity. Proc Natl Acad Sci U S A.

[CR47] Weiss S (2015). Edibles: for experts only? Ingesting marijuana, as opposed to smoking it, has come a long way since the days of homemade pot brownies. State Legis.

[CR48] Zhu B, Guo H, Cao Y, An R, Shi Y (2021). Perceived Importance of Factors in Cannabis Purchase Decisions: A Best-worst Scaling Experiment. Int J Drug Policy.

