# Supplementary material for: Oral Cannabis consumption and intraperitoneal THC:CBD dosing results in changes in brain and plasma neurochemicals and endocannabinoids in mice
Source: J Cannabis Res. 2024 Mar 2;6:10. doi: 10.1186/s42238-024-00219-x (PMC10908076; doi:10.1186/s42238-024-00219-x)
Supplement: Supplementary file 1 — Supplementary Material 1. [file 42238_2024_219_MOESM1_ESM.docx]

**Supplemental File 1: Supplemental Results, Figures, and Tables**

**Oral *Cannabis* consumption and intraperitoneal THC:CBD dosing results in changes in brain and plasma neurochemicals and endocannabinoids in mice.**

Nichole Reisdorph^1^, Katrina Doenges^1^, Cassandra Levens^2^, Jon Manke^1^, Michael Armstrong^1^, Harry Smith^1^, Kevin Quinn^1^, Richard Radcliffe^1^, Richard Reisdorph^1^, Laura Saba^1^, Kristine Kuhn^2^

^1^Skaggs School of Pharmacy and Pharmaceutical Sciences, University of Colorado Anschutz Medical Campus, Aurora, CO 80045

^2^School of Medicine, University of Colorado Anschutz Medical Campus, Aurora, CO 80045

Corresponding Author: Nichole Reisdorph email: Nichole.Reisdorph@CUAnschutz.edu

**Supplemental Results:**

**General Health and Body Weight of Mice**

As described in the Methods section, the study was conducted across two experiments to maximize the use of a limited number of telemetry devices. Mice in both groups remained healthy throughout the study, with no noted changes in food or water intake, activity, or other health measures due to treatment. All mice gained weight over the course of the study as expected due to their age, regardless of the treatment group or dose (p < 0.001). The variation was largely due to within subject variation, accounting for up to 77.43% of variation. A slight interaction effect between dose and time was seen with the Experiment 1 (January) i.p. group (p = 0.016). However, when weights between Experiment 1 and Experiment 2 i.p. mice were compared, no interaction effect was seen (p = 0.54) indicating the body weights between these sets of animals were not significantly different.

Toxicity: Toxicity was evaluated for mice receiving the control and high doses of CBD:THC by i.p. and for mice receiving control, medium, and high doses of whole Cannabis via free feeding. Measurements were conducted by the Shared Pathology Core at the University of Colorado. A sample size of n=2 mice per treatment/dose were used and some samples (FF control, IP high, and IP control) were pooled due to low sample volume. For all treatments and doses, creatinine measurements were either below or within reference ranges (0.5-1.6 mg/dl). Total protein was within reference ranges (5-7 g/dl) with the exception of the pooled FF control, which was 10% higher than the reference range. Values for ALT were all within reference ranges (10-190 U/I). Results for ALP, ALB and TBIL were unreliable due to presence of hemolysis (samples were not centrifuged in a timely manner due to time it took to harvest mice)

**LC/MS/MS of Cannabis to Determine CBD and THC Concentration**

Whole leaf Cannabis, described as very high CBD (>10%), low THC (<1%) Cannabis from NIDA was heated and prepared as described in methods. Following LC/MS/MS it was determined that 1.208 mg CBD and 0.029 mg of THC was present in 50 mg of Cannabis. This is the equivalent of 0.024 mg CBD/mg Cannabis and 0.00058 mg THC/mg Cannabis, or 3.3% CBD and 0.12% THC by weight. The ratio of CBD:THC was therefore 41:1.

**Supplemental Figures:**


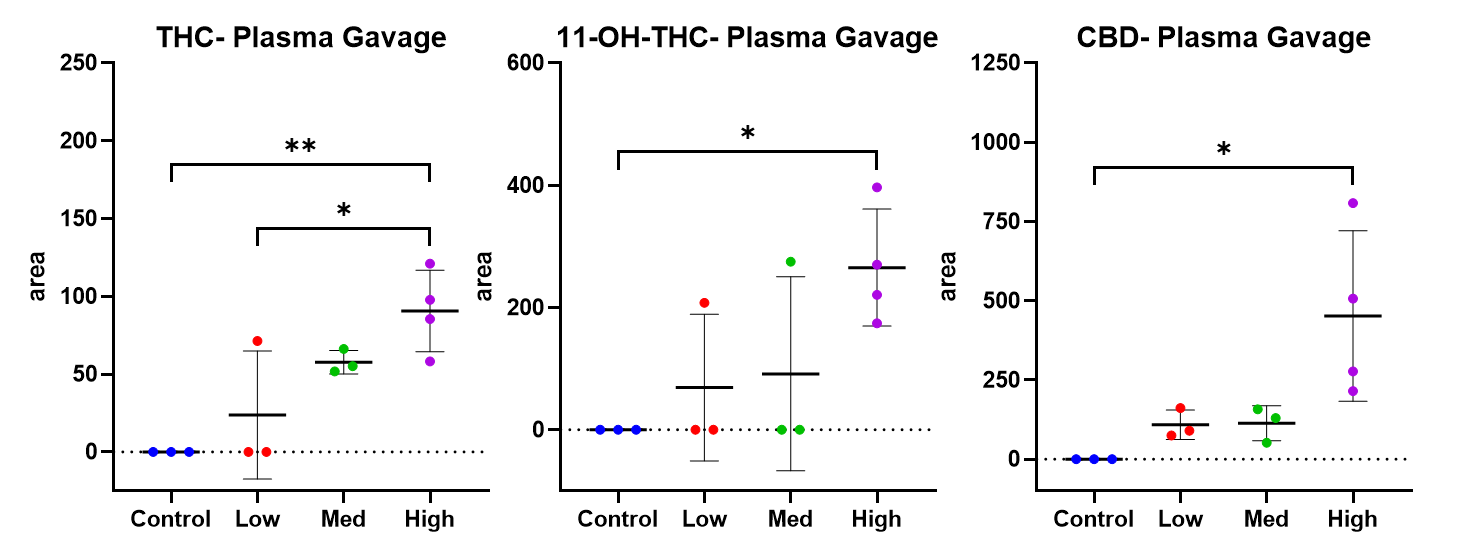


**Supplemental Figure 1:** **Significant changes (ANOVA *p*<0.05) in plasma levels of Δ9-THC, 11-OH-THC and CBD were quantified following gavage of THC/CBD (*n*=3,4).** Significance thresholds are shown following Tukey’s post hoc t-test analysis (*p < 0.05, **p < 0.01). THC was dosed at 10 mg/kg/day for 14 days while CBD was given at 10 (Low), 50 (Med), and 100 (High) mg/kg/day.


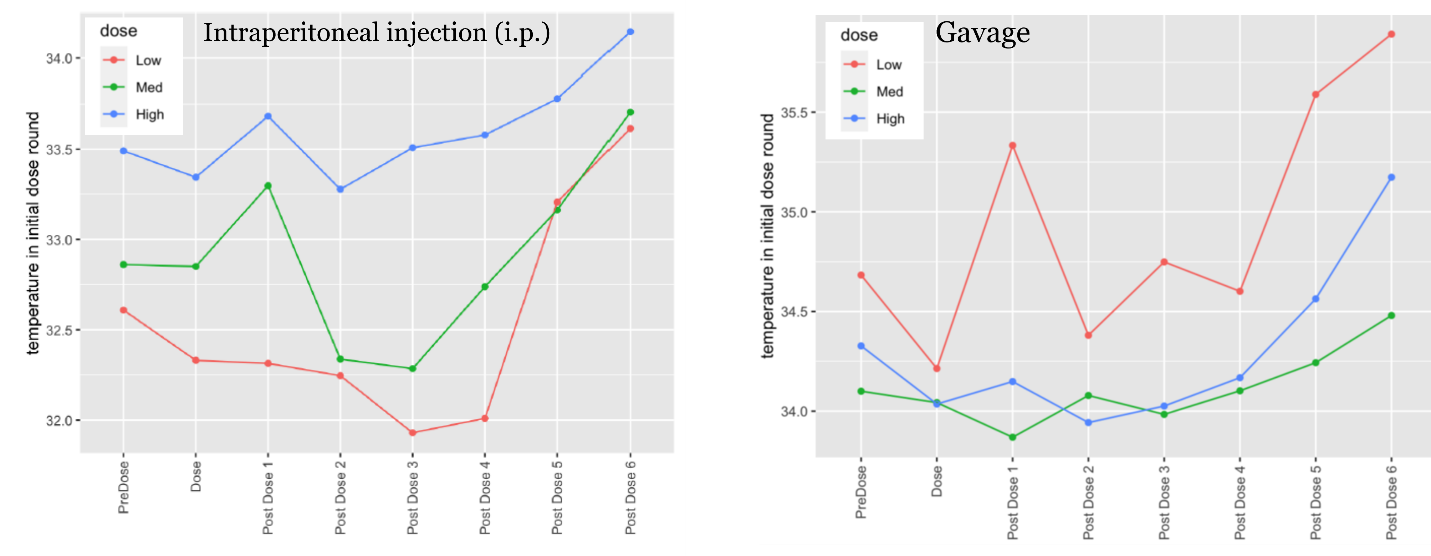


**Supplemental Figure 2:** **Acute effects on body temperature following initial exposure:** Comparison of 1-hour time windows on the first day of treatment was used to examine acute effects on body temperature during initial exposure to *Cannabis*. Acute effects were seen with i.p. (dose p = 0.03 and time p < 0.001) administration of THC/CBD; however, the effect of time on temperature did not differ between doses. There were significant differences in pre-dose temperatures in the i.p. treated mice (p = 0.013); while an explanation for this was not apparent, results should be interpreted with caution. Time and dose-dependent, acute effects were seen with administration of a complex Cannabis extract by gavage (time p = <0.001 and interaction effect p = 0.025). There were no significant differences in pre-dose temperature between the gavage groups (p = 0.906). No acute effects to body temperature were seen with the mice who were free-fed Cannabis.

**Supplemental Tables:**

| Compound | Name | Precursor Ion | Product Ion | Collision Energy |
| --- | --- | --- | --- | --- |
| THC | (-)-Δ9-THC | 315.2 | 193.2 | 29 |
| CBD | Cannabidiol | 315.23 | 193 | 25 |
| 11-OH-THC | (±)-11-Hydroxy-Δ9-THC | 331.2 | 313.3 | 13 |
| CBD-D3 | Cannabidiol-D3 | 318.2 | 196.3 | 25 |
| THC_D3 | (-)-Δ9-THC-D3 | 318.2 | 196.1 | 25 |
| 11-OH-THC-D3 | (±)-11-Hydroxy-Δ9-THC-D3 | 334.25 | 316.2 | 13 |

**Supplemental Table 1: Cannabinoid MRM transitions.** Quantitation of cannabinoids was performed using LC/MS/MS as described in the main text. All compounds were optimized and analyzed with a cell accelerator voltage of 4 and a fragment voltage of 380.

| Name | Precursor Ion | Product Ion | Collision Energy | ISTD Compound Name |
| --- | --- | --- | --- | --- |
| Melatonin | 233.1 | 174.1 | 13 | Adenosine-(ribose-13C5) |
| Niacinamide (NAM) | 123.1 | 80.1 | 20 | Adenosine-(ribose-13C5) |
| Pyroxidal | 168.1 | 106.0 | 21 | Adenosine-(ribose-13C5) |
| Isopyridoxal | 168.1 | 122.0 | 16 | Adenosine-(ribose-13C5) |
| Quinolinic Acid | 168.0 | 150.1 | 5 | Adenosine-(ribose-13C5) |
| 3,4-DiOHPheAc Acid | 169.1 | 123.1 | 9 | Adenosine-(ribose-13C5) |
| Adenosine-ribose-13C5 (ISTD) | 273.1 | 136.2 | 25 | <None> |
| Creatinine-d3 (ISTD) | 117.1 | 89.0 | 9 | <None> |
| Anthranilic Acid | 138.1 | 120.1 | 9 | Creatinine-d3 |
| Kynurenic acid | 190.0 | 144.0 | 16 | Creatinine-d3 |
| Metanephrine | 198.1 | 180.1 | 5 | Creatinine-d3 |
| Epinephrine | 184.1 | 166.0 | 5 | U13C-Leucine |
| 3-hydroxy-athranilic acid (3-HAA) | 154.0 | 80.1 | 28 | U13C-Phenylalanine |
| Serotonin | 177.1 | 160.0 | 8 | U13C-Phenylalanine |
| N-Methylnicotinamide | 137.1 | 94.0 | 21 | U13C-Phenylalanine |
| Phenylalanine | 166.1 | 120.1 | 8 | U13C-Phenylalanine |
| Dopamine | 154.1 | 91.0 | 24 | U13C-Phenylalanine |
| U13C-Phenylalanine (ISTD) | 175.0 | 128.0 | 12 | <None> |
| Xanthurenic Acid | 206.1 | 160.0 | 16 | U13C-Phenylalanine |
| L-Kynurenine | 209.1 | 94.0 | 13 | U13C-Phenylalanine |
| U13C-Leucine (ISTD) | 138.0 | 91.0 | 8 | <None> |
| Normetanephrine | 184.1 | 166.1 | 5 | U13C-Leucine |
| Picolinic Acid (PA) | 124.0 | 78.0 | 20 | U13C-Leucine |
| N-Formylkynurenine | 237.1 | 146.1 | 24 | U13C-Leucine |
| Tryptophan | 205.1 | 188.1 | 4 | U13C-Leucine |
| Norepinephrine (M-H2O)H+ | 152.1 | 77.0 | 29 | U13C-Leucine |
| 5-Hydroxyindole-3-acetic acid (5-HIAA) | 192.1 | 146.1 | 13 | U13C-Leucine |
| Glycyl-Phenylalanine | 223.1 | 120.0 | 20 | U13C-Leucine |
| 3-Hydroxy Kynurenine | 225.1 | 110.0 | 17 | U13C-Leucine |
| U13C-Proline (ISTD) | 121.0 | 74.0 | 16 | <None> |
| Tyrosine | 182.1 | 91.1 | 32 | U13C-Proline |
| NR-d2 (ISTD) | 257.1 | 124.0 | 8 | <None> |
| 5-Hydroxytryptophan | 221.1 | 204.0 | 5 | NR-d2 |
| Arginyl-Phenylalanine | 322.2 | 70.1 | 40 | U13C-Glutamine |
| U13C-Glutamine (ISTD) | 152.0 | 88.0 | 16 | <None> |
| GABA | 104.1 | 87.0 | 9 | U13C-Glutamine |
| NAD+U13C (ISTD) | 685.2 | 141.0 | 48 | <None> |
| NAD+ | 664.1 | 135.9 | 48 | NAD+(U13C) |

**Supplemental Table 2: Neurochemical MRM transitions.** Quantitation of neurochemicals was performed using LC/MS/MS as described in the main text. All compounds were optimized and analyzed with a cell accelerator voltage of 4 and a fragmentor voltage of 380.

| Name | Precursor Ion | Product Ion | Collision Energy | ISTD Compound Name |
| --- | --- | --- | --- | --- |
| Eicosapentaenoyl Ethanolamide (EPEa) | 346.3 | 62.1 | 14 | Arachidonoyl Ethanolamide-d4 (AEA-d4) |
| Palmitoleoyl Ethanolamide (POEA) | 298.3 | 62.1 | 18 | Arachidonoyl Ethanolamide-d4 (AEA-d4) |
| Docosahexaenoyl Ethanolamide (DHEa) | 372.3 | 62.1 | 14 | Arachidonoyl Ethanolamide-d4 (AEA-d4) |
| Arachidonoyl Ethanolamide-d4 (AEA-d4 ISTD) | 352.3 | 66.0 | 14 | <None> |
| Arachidonoyl Ethanolamide (AEA) | 348.3 | 62.0 | 18 | Arachidonoyl Ethanolamide-d4 (AEA-d4) |
| Linoleoyl Ethanolamide (LEA) | 324.3 | 62.0 | 14 | Arachidonoyl Ethanolamide-d4 (AEA-d4) |
| 2-Arachidonoyl Glycerol-d5 (2-AG-d5 ISTD) | 384.3 | 287.3 | 10 | <None> |
| Arachidonoyl Glycine (NAGly) | 362.3 | 287.2 | 10 | 2-Arachidonoyl Glycerol-d5 (2-AG-d5) |
| 2-Arachidonoyl Glycerol (2-AG) | 379.3 | 287.2 | 10 | 2-Arachidonoyl Glycerol-d5 (2-AG-d5) |
| 2-Linoleoyl Glycerol (2-LG) | 355.3 | 263.2 | 6 | 2-Arachidonoyl Glycerol-d5 (2-AG-d5) |
| Palmitoyl Ethanolamide (PEA) | 300.3 | 62.0 | 14 | Oleoyl Ethanolamide-d4 (OEA-d4) |
| Oleoyl Ethanolamide-d4 (OEA-d4 ISTD) | 330.3 | 66.0 | 20 | <None> |
| Oleoyl Ethanolamide (OEA) | 326.3 | 62.0 | 14 | Oleoyl Ethanolamide-d4 (OEA-d4) |
| Docosatetraenoyl Ethanolamide (DEA) | 376.3 | 62.1 | 18 | Oleoyl Ethanolamide-d4 (OEA-d4) |
| Stearoyl Ethanolamide (SEA) | 328.3 | 62.1 | 14 | Oleoyl Ethanolamide-d4 (OEA-d4) |

**Supplemental Table 3: Endocannabinoid MRM transitions.** Quantitation of neurochemicals was performed using LC/MS/MS as described in the main text. For MRM transitions, all compounds were optimized and analyzed with a cell accelerator voltage of 2 and a fragmentor voltage of 380.

**Please also see the following Supplemental Files:**

**Supplemental File 2**: Levels of THC, CBD, and 11-OH-THC in plasma and brains of mice following treatment. “Cannabinoids Summary Data.xlsx”. Cells in green are considered significant at p-value < 0.05.

**Supplemental File 3**: Levels of neurochemicals in plasma and brains of mice following treatment and ANOVA results. “Summary Neurotransmitters Data.xlsx”. Cells in green are considered significant at p-value < 0.05.

**Supplemental File 4**: Levels of endocannabinoids in plasma and brains of mice following treatment and t-test or ANOVA results. “Summary Endocannabinoid Data.xlsx” Cells in green are considered significant at p-value < 0.05.
